# GRAIL and Otubain-1 are Related to T Cell Hyporesponsiveness during *Trypanosoma cruzi* Infection

**DOI:** 10.1371/journal.pntd.0005307

**Published:** 2017-01-23

**Authors:** Cinthia C. Stempin, Jorge D. Rojas Marquez, Yamile Ana, Fabio M. Cerban

**Affiliations:** Centro de Investigaciones en Bioquímica Clínica e Inmunología (CIBICI), CONICET, Departamento de Bioquímica Clínica, Facultad de Ciencias Químicas, Universidad Nacional de Córdoba, Medina Allende y Haya de la Torre, Ciudad Universitaria, Córdoba, Argentina; New York University, UNITED STATES

## Abstract

**Background:**

*Trypanosoma cruzi* infection is associated with severe T cell unresponsiveness to antigens and mitogens and is characterized by decreased IL-2 synthesis. In addition, the acquisition of the anergic phenotype is correlated with upregulation of “gene related to anergy in lymphocytes” (GRAIL) protein in CD4 T cells. We therefore sought to examine the role of GRAIL in CD4 T cell proliferation during *T*. *cruzi* infection.

**Methodology/Principal Findings:**

Balb/c mice were infected intraperitoneally with 500 blood-derived trypomastigotes of Tulahuen strain, and spleen cells from control non-infected or infected animals were obtained. CD4 T cell proliferation was assessed by CFSE staining, and the expression of GRAIL in splenic T cells was measured by real-time PCR, flow cytometry and Western blot. We found increased GRAIL expression at the early stages of infection, coinciding with the peak of parasitemia, with these findings correlating with impaired proliferation and poor IL-2 and IFN-γ secretion in response to plate-bound antibodies. In addition, we showed that the expression of GRAIL E3-ubiquitin ligase in CD4 T cells during the acute phase of infection was complemented by a high expression of inhibitory receptors such as PD-1 and CTLA-4. We demonstrated that GRAIL expression during infection was modulated by the mammalian target of the rapamycin (mTOR) pathway, since addition of IL-2 or CTLA-4 blockade in splenocytes from mice 21 days post infection led to a reduction in GRAIL expression. Furthermore, addition of IL-2 was able to activate the mTOR pathway, inducing Otubain-1 expression, which mediated GRAIL degradation and improved T cell proliferation.

**Conclusions:**

We hypothesize that GRAIL expression induced by the parasite may be maintained by the increased expression of inhibitory molecules, which blocked mTOR activation and IL-2 secretion. Consequently, the GRAIL regulator Otubain-1 was not expressed and GRAIL maintained the brake on T cell proliferation. Our findings reveal a novel association between increased GRAIL expression and impaired CD4 T cell proliferation during *Trypanosoma cruzi* infection.

## Author Summary

Chagas disease is caused by the protozoan parasite *Trypanosoma cruzi* and is endemic in Central and South America, where it affects about 10 million people. In addition, migration has led to the disease being established in non-endemic countries. Infection involves an acute stage that evolves to a chronic stage where infected individuals may or may not show clinical symptoms or suffer progressive heart disease. The relevance of T cells in the control of *T*. *cruzi* infection has been demonstrated in human infection and in experimental models. However, the *T*. *cruzi* parasite employs different strategies to downregulate the T cell function. These mechanisms can act at the initial time of T cell activation, leading to a state of anergy where lymphocytes do not respond. However, the molecular components that regulate this process during *T*. *cruzi* infection are not well understood. Our findings demonstrate for the first time that this T cell hyporesponsiveness could be linked to an increased expression of GRAIL. We propose that GRAIL expression induced by the parasite could be maintained by increased expression of inhibitory molecules, which blocked mTOR activation and IL-2 secretion. GRAIL could then play a key role in downregulating T cell functions by allowing the parasites to establish the chronic disease.

## Introduction

Chagas disease, caused by the intracellular protozoa *Trypanosoma cruzi*, is one of the major human health problems in Latin America. It evolves from an acute to a chronic phase, where subjects may be clinically asymptomatic or show progressive heart disease and leads to an end-stage dilated cardiomyopathy in 20–30% of infected individuals. It is estimated that approximately 4 million chagasic individuals have developed heart disease, making Chagas disease the most frequent cause of infectious cardiomyopathy in the world [1,2].

The immune control of *T*. *cruzi* is complex, requiring the generation of a substantial antibody response and the activation of both CD4 and CD8 T cell responses. Even in cases in which these responses are sufficiently stimulated to be able to control the acute infection, *T*. *cruzi* is not completely eradicated, but instead persists in infected hosts for decades [3].

*T*. *cruzi* employs a variety of strategies to evade the immune system and remain in the infected host. The main method involves the inhibition of specific T-cell responses, and consequently, can favor the establishment of chronic infections [4,5,6,7,8]. Related to this, a number of both host-dependent and parasite-induced mechanisms have been previously shown to affect immune regulation [9,10]. Moreover, T cells from infected hosts are largely unresponsive to antigens and mitogens, resulting in reduced IL-2 synthesis [8].

IL-2 production initiates proliferation, effector functions, and clonal expansion via IL-2 receptor (IL-2R)-mediated signaling [11]. In the absence of a robust activation initiated by TCR and CD28 signaling, CD4 T cells fail to proliferate or to produce IL-2 and enter a state of unresponsiveness following immunogenic stimulation, referred to as “anergy” [11,12]. In the case of CD4 T cells, the development of anergy depends on the alteration of the expression of several genes [11,12,13].

Post-translational modification of proteins via ubiquitination also plays an essential role in the regulatory mechanism of CD4 T cell anergy [14,15]. GRAIL, also known as ring finger protein-128 (RNF-128), has been identified as a novel E3 ubiquitin-protein ligase that induces and maintains anergy in CD4 T cells [16,17,18]. It has been shown that GRAIL expression could be correlated with the inhibition of CD4 T cell proliferation and antigen-induced IL-2 transcription by disrupting the T cell stimulatory signaling [19]. In support of this observation, T cells from GRAIL knock-out mice were shown to be defective in anergy induction both *in vitro* and *in vivo* [17,20]. In particular, GRAIL (-/-) T cells hyperproliferated [17,21] and produced more cytokines [17] compared with wild type (WT) cells in response to TCR stimulation alone *in vitro* or with concomitant anti-CD28 costimulation. It has also been recently demonstrated that GRAIL, by mediating TCR-CD3 degradation, regulated naive T cell tolerance induction [20]. Furthermore, several investigations have shown how GRAIL interacts with T-cells, antigen presenting cell (APC) receptors and cytoskeletal proteins, thereby promoting their degradation [22,23,24,25].

GRAIL expression is regulated by Otubain-1 (Otub-1) [26], which is a member of deubiquitinating enzymes with the capability to cleave proteins at the ubiquitin-protein bond by using its cysteine protease domain [27]. It has been shown that Otub-1 is expressed and GRAIL degraded when naive CD4 T cells are productively activated to undergo proliferation [19]. Moreover, the loss of GRAIL was mechanistically controlled through a pathway involving CD28 co-stimulation, IL-2 production and IL-2R signaling, and ultimately, mTOR-dependent translation of select mRNAs. Blocking the mTOR by using CTLA-4-Ig, anti-IL-2 or rapamycin prevented Otub-1 protein expression and maintained GRAIL expression, which inhibited T cell proliferation [19]. Although the function of GRAIL in CD4 T cells has been studied extensively for the development of tolerance [17,20], with its participation having been demonstrated in the development of autoimmune diseases [20], only recently has its role been studied during T cell dysfunction in the course of infections [28,29,30,31]. Thus, the aim of this work was to search for a novel link between GRAIL and CD4 T cell unresponsiveness in the context of abnormalities of T cell proliferation observed during *Trypanosoma cruzi* infection. Our results provide evidence demonstrating that CD4 T cells from *T*. *cruzi* infected mice exhibited an increase in GRAIL expression during the acute phase of infection, which was correlated with defects in proliferation and immune responsiveness. In addition, we showed that high expression of CTLA-4 and low levels of IL-2 prevented mTOR activation and Otub-1 protein expression, and maintained GRAIL expression, which inhibited T cell proliferation during the acute phase of the infection. Our results therefore indicate that GRAIL is an important player in CD4 T cell anergy during the acute phase of *Trypanosoma cruzi* infection.

## Methods

### Ethics statement

All the animal experiments were examined by the Institutional Experimentation Animal Committee, from Facultad de Ciencias Químicas, Univesidad Nacional de Córdoba, which approved the experimental procedures (authorization no. 2016–209). This committee follows the guidelines for animal care of “Guide to the care and use of experime**n**tal animals” (Canadian Council on Animal Care, 1993) and of “Institutional Animal Care and Use Comittee Guidebook” (ARENA/OLAW IACUC Guidebook, Nacional Institutes of Health, 2002).

### Mice and *T*. *cruzi* infection

BALB/c mice obtained from the Comisión Nacional de Energía Atómica (CNEA; Buenos Aires, Argentina) were inbred and housed according to institutional guidelines. BALB/c mice, when 6–8 weeks old, were intraperitoneally infected with 1x10^6^ blood-derived *T*. *cruzi* trypomastigote forms from Tulahuén strain, which was maintained through intraperitoneal inoculation every 11 days [32].

Female BALB/c mice were infected intraperitoneally with 500 blood-derived *T*. *cruzi* trypomastigote forms diluted in saline solution. After different days post infection (p.i.), these mice were sacrificed by CO_2_ asphyxiation and spleens were extracted. Non-infected animals were processed in parallel. Trypomastigotes of the Tulahuen and Y strains were obtained from the extracellular medium of infected monolayers of Vero or NIH3T3 cells, respectively, and were collected by centrifugation at 4400 rpm for 10 min and resuspended in RPMI medium containing 10% FCS. Parasites were counted using a Neubauer chamber and used for *in vitro* infection experiments as described below.

### Isolation of splenocytes

Spleen cells were obtained from control or infected animals by homogenizing the organs in a cell strainer. Then, the spleen cell suspensions were centrifuged (1500 rpm, 5 min, 4°C) and the pellets treated with RBC lysis buffer (GIBCO). These cells were subsequently resuspended in complete RPMI medium containing 10% fetal bovine serum (FBS, PAA laboratories), L-glutamine (2 mM, GIBCO) and gentamicin (40 g/ml), and the isolated cell suspensions were passed through a 50-mm nylon mesh (BD Falcon) for cell culture, flow cytometry or cell isolation. Finally, the T cells were isolated from spleens using a CD4+ T cell isolation kit, according to the manufacturer’s instructions (Miltenyi Biotec), with the average purity found to be 95–98%.

### HEK-293 cell line

HEK-293 cells (ATCC) were maintained in Dulbecco's modified Eagle's medium (Gibco) supplemented with 10% fetal bovine serum (FBS, PAA laboratories), L-glutamine (2 mM, GIBCO) and gentamicin (40 g/ml), and these cells were grown at 37°C under 5% CO_2_.

### Fluorescence-activated cell-scanning (FACS) analysis

To examine PD-1, CTLA-4 and GRAIL expression, splenocytes or CD4 T cells from control and infected animals or *in vitro* infected CD4 T cells were washed with saline solution 2% FBS and incubated with anti-mouse CD32/CD16 antibody for 20 minutes at 4°C to block Fc receptors. Then, cells were incubated with APC labeled anti-CD4, PercP labeled anti-CD3 (BD Pharmingen), and with PE-labeled anti-PD-1 or anti-CTLA-4 (BD Pharmingen) for 20 min at 4°C.

For the assessment of intracellular GRAIL expression, cells were first stained with FITC-CD3 and APC-CD4 antibodies, and then fixed and permeabilized with Citofix/Citoperm (BD Biosciences,) for 30 min followed by reacting with rabbit anti-GRAIL primary Ab (Abcam) for 45 min. After washing, cells were stained for 20 min with PE–anti-rabbit IgG (Biolegend). Finally, cells were washed twice with saline solution of 2% FBS, and stored at 4°C in the dark until analysis using a FACS flow cytometer (FACS Canto II, BD Biosciences). The results were processed using Flow Jo software (version 7.6.2).

### Cytokine determination

Cytokines were measured in culture supernatants using a capture enzyme-linked immunosorbent assay (ELISA). IFN-γ (eBioscience) and IL-2 (Biolegend) were used as paired monoclonal antibodies in combination with recombinant cytokine standards. All assays were performed according to the manufacturer’s guidelines.

### Immunoblot analysis

CD4 T cells from control and infected animals were washed and lysed for 30 min at 4°C in RIPA buffer [1% Triton X-100 (v/v), 0.5% sodium deoxicolate (p/v), 0.1% sodium dodecyl sulfate (SDS)] containing a protease inhibitor cocktail (Roche), and the cell debris was spun down at 13,000 g for 15 min. Precipitates were removed, and aliquots of the cell lysates were diluted in SDS sample buffer, boiled at 100°C for 3 min, spun down, and applied to precast 10% acrylamide Tris-glycine gels at 40 μg protein/lane and run at 150 V for 1 h. Samples were transferred to nitrocellulose membranes (BioRad) at 100 V for 1 h, and these membranes were probed using rabbit anti-mouse GRAIL (Santa Cruz Biotechnology), anti-mouse Otub-1 or anti-mouse p-4EBP1 (Cell Signaling Technology) followed by peroxidase conjugated anti-rabbit antibody (Sigma Chemical Co.), before being visualized using enhanced chemiluminescence (Pierce) for detection. The protein loading was evaluated by actin expression.

### Quantitative real-time PCR

RNA was extracted from splenocytes from infected or control animals by the Trizol reagent (Invitrogen) and reverse-transcribed into cDNA by using Revert Aid First Strand cDNA Synthesis (Fermentas). Transcripts were quantified by real-time quantitative PCR on an ABI Prism 7500 sequence detector (Applied Biosystems) with predesigned TaqMan gene expression assays and reagents (Applied Biosystems), according to the manufacturer´s instructions. Probes with the following Applied Biosystems assay identification numbers were used: Cblb, Mn01343092.m1; Rnf128, Mn00480990.m1; Rn18s, Mn03928990.g1. For each sample, mRNA abundance was normalized to the amount of 18S RNA and expressed as arbitrary units.

### Cell proliferation assay

CD4 T cell proliferation was measured using the cell division tracking dye carboxyfluorescein diacetate succinimidyl ester (CFSE) (Molecular Probes, Eugene, OR). Spleen CD4 T cells isolated from infected or control animals were stained with CFSE dye at 5 μM concentrations. Cells were incubated at 37°C for 10 min, and then the reaction was stopped by adding 10 ml of RPMI medium containing 10% FBS. After washing, cells were resuspended in warm RPMI complete medium before being plated in anti-CD3/CD28 Abs (1 μg/mL of each Abs)-coated plates. After 72 h of incubation, cells were stained with PerCP/Cy5.5-CD4 Ab and acquired for FACS analysis. Unstimulated CFSE-labeled cells served as a non-dividing control. Data analysis was performed using a FACS flow cytometer (FACS Canto II, BD Biosciences) with FlowJo software (version 7.6.2), by setting a gate on the live cells to side-scatter versus forward-scatter dot plots and determining the expression of the CFSE.

### *In vitro* stimulation of recombinant mouse IL-2 (rmIL-2)

CD4+ T cells were isolated from the spleen of control or infected animals and then cultured in complete RPMI with or without rmIL-2 (R&D Systems) at a concentration of 20 ng/mL for 3 days for the cell proliferation assays, or for 1 day for the assessment of GRAIL intracellular expression.

### *In vitro* blockade of CTLA-4

Splenocytes were isolated at 21 days p.i. and then cultured in complete RPMI medium in the presence of CTLA-4 blocking antibody or control antibody (10 μg/ml, eBioscience). Then, intracellular GRAIL expression was evaluated 48 h later by flow cytometry.

### Statistical analysis

Statistical analyses were performed using the student’s t-test. Values of p < 0.05 were considered to be statistically significant.

## Results

### CD4 T cells from *T*. *cruzi* infected mice exhibit impaired proliferation and cytokine production during the acute phase

**Fig 1:**
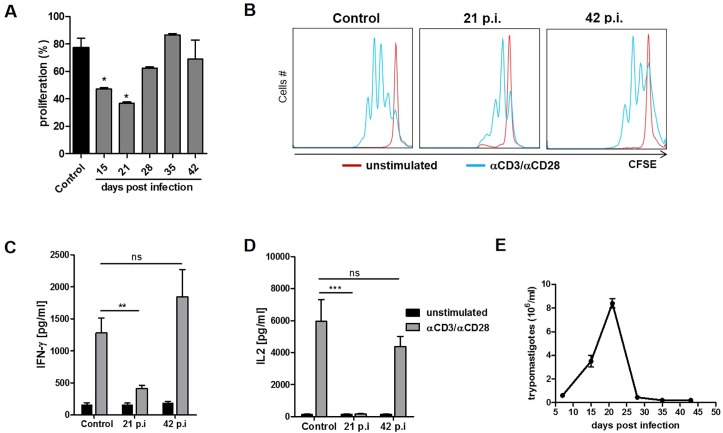
Proliferation and cytokine production are impaired in CD4 T cells during the acute phase of *T*. *cruzi* infection. CD4+ T cells from spleen control or infected animals at various days post-infection were purified by magnetic beads. Then, cells were stained with CFSE and cultured in 96-well plates with bound anti-CD3 (1 ug/ml) plus CD28 (1 ug/ml). After 72 h incubation, proliferation was assessed by flow cytometry, and cytokine production was evaluated by ELISA. CD4+ T cell proliferation at different times post-infection was compared to proliferation in control cells and quantitatively analyzed as a percentage of proliferation by FlowJo software (p< 0.05 * versus control) (A). A representative histogram with CFSE dye dilution in dividing cells is shown (B). Supernatants were harvested and the amount of IFN-γ (C) and IL-2 (D) were determined by ELISA sandwich **p = 0.0044, ***p = 0.0001, paired t- test versus control. Parasitemia was determined at different time points post *T*. *cruzi* infection (E), n = 6. IFN-γ, IL-2 and the proliferation data are the mean ± SEM of three independent experiments (n = 4 mice/group).

To analyze the proliferative efficacy of the CD4 T cells, they were first isolated from the spleen of control and *T*. *cruzi* infected mice at different time points. Then, cells were stained with CFSE, stimulated with anti-CD3/CD28, and proliferation was analyzed 72 h later. CD4 T cells isolated from the spleen of infected animals showed a considerable decrease in their proliferation during the acute phase of infection compared to CD4 T cells from control animals (Fig 1A and 1B). However, proliferation of CD4 T cells from infected animals was recovered later on in the infection. In addition, stimulated CD4 T cells from infected animals produced less IFN-γ and IL-2 at an early time point of infection (21 p.i.) compared to control CD4 T cells (Fig 1C and 1D). These results coincided with the peak of parasitemia (Fig 1E).

### CD4 T cells from *T*. *cruzi* infected mice express inhibitory receptors

Because CTLA-4 and PD-1 are known to inhibit T cell function, we examined the expression levels of these molecules in cells from the spleens of control and infected animals.

**Fig 2:**
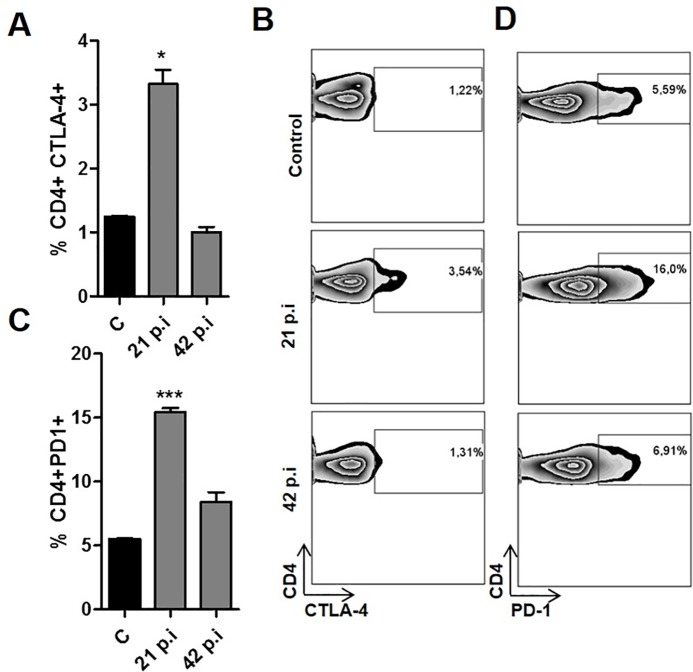
Increased expression of inhibitory molecules on CD4 T cells in the acute phase of *T*. *cruzi* infection. Spleen cells isolated from control and infected animals were stained with anti-CD4, anti-CD3, anti-PD-1 or anti-CTLA-4 mAbs. Then, cells were analyzed by flow cytometry. Graphs show the percentages of CD4+ CTLA-4+ (A, * p< 0.05 versus control) or CD4+ PD1+ cells (C, *** p< 0.0001 versus control). The data are mean ± SEM (n = 3) with representative dot plot graphs of at least three independent experiments being shown (B and D).

It has been shown that *T*. *cruzi* is able to modulate the expression levels of the negative coreceptor PD-1 in several immune cells [33]. However, in that study, different mice and parasite strains were used. Thus, to evaluate in our experimental model if *T*. *cruzi* infection upregulates PD-1 and CTLA-4 expression in CD4 T cells, flow cytometry was performed on spleen cells at several time points after infection, and the percentage of CD4+ T cells expressing PD-1 or CTLA-4 on the surface was determined, as shown in Fig 2A and 2C, with representative dot plots displayed in Fig 2B and 2D. We found that the infection led to an increase in the expression of PD-1 and CTLA-4 in spleen CD4 T cells from infected mice at 21 days p.i. compared to control cells (Fig 2A and 2C). In addition, we found that expression levels of CTLA-4 fell to normal levels at day 42 p.i., and PD-1 expression also decreased significantly (Fig 2B and 2D).

### GRAIL is upregulated in CD4 T cells during the acute phase of infection

**Fig 3:**
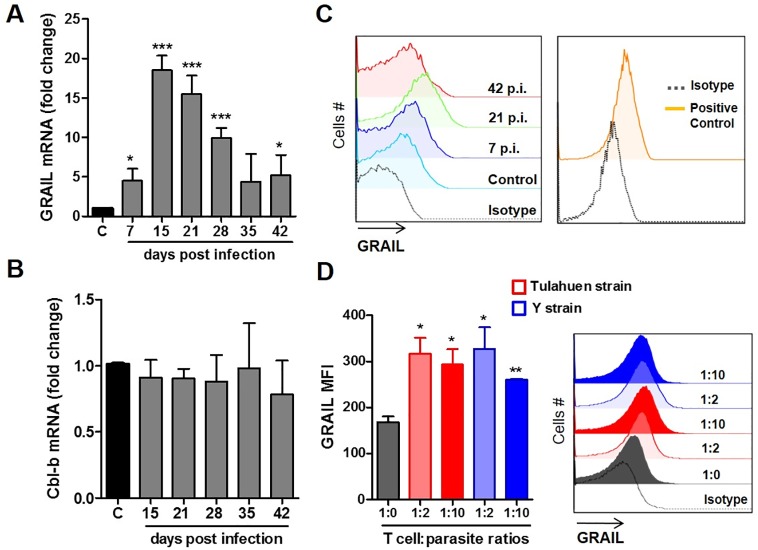
Increased GRAIL E3-ubiquitin ligase expression in spleen T cells during *T*. *cruzi* infection. Cells were isolated from spleens of control or infected animals at different time points after infection, then these cells were used to extract mRNA and subsequent real-time PCR was performed to analyze GRAIL (RNF128) (A) or Cbl-b (B) expression. The expression of 18S served as an internal control and the data are expressed as fold induction compared to control animals. This experiment is representative of two independent experiments (*** p <0.0001; * p <0.05). GRAIL expression was evaluated by FACS in spleen cells isolated from control and infected animals at different days p.i. (C) and in CD4 T cells *in vitro* cultured with two different *T*. *cruzi* strains at different T cell/parasite ratios (D). Additionally, GRAIL expression in HEK 293 cells was used as a positive control (C). Then, 2x10^6^ cells were stained with anti-CD4 and anti-CD3 antibodies. After cells were fixed and permeabilized, GRAIL expression was detected using an anti-GRAIL antibody followed by an anti-rabbit IgG-PE. Cells were analyzed by flow cytometry on CD3+CD4+ gated cells. Representative histograms are shown (C and D) and bars display GRAIL mean fluorescence intensity (MFI, D) (n = 4, *p <0.05; **p = 0.0019).

Considering that GRAIL is an inducer of impaired CD4 T cell proliferation during *in vitro* and *in vivo* tolerance [16,17], as well as being involved in CD4 T cell dysfunction during infection [28,29,30], we aimed to assess its expression in spleen cells from control and infected animals by real time PCR. We found a significant upregulation of its mRNA levels in splenocytes from infected mice, which were strongest at the earliest time point post-infection (Fig 3A). However, we did not observe upregulation of Cbl-b, which is another E3-Ubiquitin ligase shown to be involved in regulating T cell functions (Fig 3B) [34,35,36]. To confirm GRAIL expression in CD4 T cells, splenocytes were labeled with anti-CD4 and anti-CD3 antibodies, and then GRAIL expression was evaluated by intracellular labeling and analyzed by flow cytometry. A considerable upregulation of GRAIL protein was found in splenic CD4 T cells isolated from animals at 21 days p.i., compared to splenic CD4 T cells from control uninfected animals. However, GRAIL expression decreased in cells from animals at 42 days p.i. (Fig 3C). GRAIL expression in the HEK 293 cell line was used as a positive control (Fig 3C).

Next, to test if GRAIL upregulation in CD4 T cells is a general phenomenon of *T*. *cruzi* infection or is dependent on the strain used, we evaluated GRAIL expression by FACS in CD4 T cells cultured *in vitro* with two different *T*. *cruzi* strains. We observed that both *T*. *cruzi* strains induced GRAIL expression at similar levels (Fig 3D). Thus, our results showed that GRAIL expression is induced during acute phase of infection and correlates with the peak of parasitemia and with CD4 T cell hiporesponsiveness. In addition, we observed that GRAIL expression is induced directly by different parasite strains.

### GRAIL expression during infection is regulated via the mTOR/Otubain-1 axis

**Fig 4:**
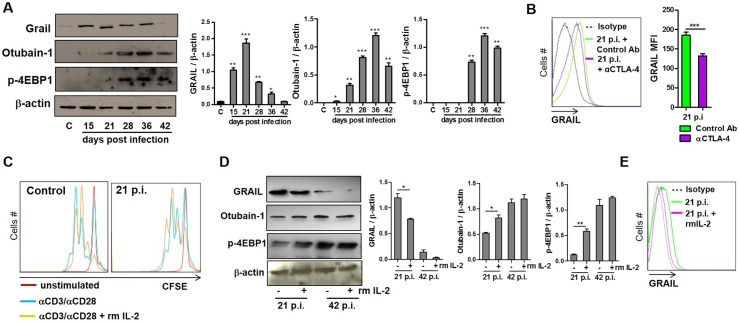
GRAIL expression is regulated by the IL2/mTOR/Otubain-1 axis in CD4 T cells during *T*. *cruzi* infection. CD4+ T cells were purified from the spleens of control or infected mice at different days post-infection. Then, cells were lysed and the expression of GRAIL, and Otub-1 and the phosphorylation of 4EBP1 were determined by Western blot using specific antibodies, with protein loading being evaluated by actin expression (A). Right panels show the densitometric analysis using GelPro software, *n* = 2 experiments *p< 0.05; **p< 0.005; ***p< 0.001. splenocytes of 21- day-p.i. animals were cultured in the presence of CTLA-4 blocking or control antibody (10 ug/ml) and GRAIL intracellular expression was evaluated by flow cytometry on CD3+CD4+ gated cells 48 h later. Representative histograms are shown and bars display GRAIL mean fluorescence intensity (MFI) *** p = 0.0006 (B). Additionally, CD4+ T cells from the spleens of control or 21-day p.i. animals were stained with CFSE (5 mM) and then cultured for 72 h in plate-bound anti-CD3/CD28 (1 μg/ml) in the presence or absence of exogenous rmIL-2 (20 ng/ml). The proliferation was evaluated by flow cytometry analysis and representative histograms indicating the CD4 T cell proliferation are shown (C). CD4+ T cells from spleen of 21 or 42-day p.i. animals were cultured in the presence or absence of exogenous rmIL-2 (20 ng/ml), and the expression of GRAIL, Otub-1 and 4EBP1 phosphorylation were evaluated by western blot. Right panels show the densitometric analysis using GelPro software, *n* = 2 experiments *p<0.05; ** p = 0.0053; (D). Intracellular GRAIL expression was evaluated by flow cytometry in CD4+ T cells from 21-day p.i. animals cultured in the presence or absence of exogenous rmIL-2 (20 ng/ml), with representative histograms being shown in (E). The data are representative of two independent experiments (n = 3 mice/group).

It has previously been shown that Otub-1 is expressed and GRAIL is degraded when naive CD4 T cells are productively activated to undergo proliferation [19]. In addition, Lin *et al*. demonstrated that the loss of GRAIL is mechanistically controlled through a pathway involving CD28 costimulation, IL-2 production and IL-2R signaling, and ultimately, by mTOR-dependent translation of select mRNA. Interference of this pathway using CTLA-4-Ig, anti-IL-2, or rapamycin prevents Otub-1 protein expression and maintains GRAIL expression, which inhibits T cell proliferation [19]. These findings implicate Otub-1 and GRAIL as important components governing T cell unresponsiveness. Given that we observed a reduced IL-2 production and increased CTLA-4 and GRAIL expression in CD4 T cells from the acute phase of *T*. *cruzi* infected mice, we evaluated GRAIL as well as Otub-1 expression and mTOR activation in CD4 T cells from *T*. *cruzi* infected mice at different time points after infection. An increased GRAIL expression was observed during infection, which was stronger at the acute phase of infection (Fig 4A) as shown also by real time PCR and FACS (Fig 3A and 3C), while GRAIL expression dropped at 42 days p.i. (Fig 4A). In addition, Otub-1 expression was not evident early on, although it increased as the infection progressed, with a peak occurring at day 36 p.i. Finally, GRAIL expression was not observed at 42 days p.i. while Otub-1 protein expression was observed (Fig 4A), showing that GRAIL downregulation happened later on in the infection and depended on Otub-1 expression.

As CD4 T cells require CD28 costimulation and IL-2R signaling to modulate GRAIL expression, we reasoned that the mTOR pathway might also control Otub-1 and GRAIL expression. On examining mTOR activity, we did not observe phosphorylation of 4EBP1 during the acute phase of infection although it was detected later on (Fig 4A), thereby allowing Otub-1 expression and GRAIL degradation. This effect could has been related to CTLA-4 expression (Fig 2A) since it has previously been shown that CTLA4-Ig treatment blocks CD28 costimulation and the resultant IL-2 expression. This in turn inhibits the mTOR-dependent translation of mRNA transcripts, including Otub-1, thus maintaining GRAIL expression and inhibiting CD4 T cell proliferation [19]. To test this hypothesis, we performed experiments by culturing splenocytes from infected mice at 21 days p.i. with blocking anti-CTLA-4 or control antibody and GRAIL intracellular expression was evaluated 48 h later by FACS. We observed a reduction in GRAIL expression in cells from infected mice cultured with anti-CTLA-4 compared to cells treated with control antibody (Fig 4B). This might indicate that the absence of co-stimulation during the acute phase of infection due to increased expression of inhibitory molecules, (Fig 2) such as CTLA-4, which may allow GRAIL expression to be maintained via a blockade of mTOR activation.

### IL-2 improves CD4 T cell proliferation from *T*. *cruzi* infected mice by downregulating GRAIL

Taking into account that GRAIL expression during *T*. *cruzi* infection might be regulated by Otub-1 and that this depends on mTOR and IL-2 signalling, we hypothesized that the addition of exogenous IL-2 to CD4 T cells at 21 days p.i. may compensate for either diminished or delayed IL-2 production. We performed *in vitro* experiments by comparing CD4 T cell proliferation in control and in animals at 21 days p.i. after treatment with rmIL-2. CD4 T cells were stimulated with anti-CD3/CD28 ligands in the presence or absence of rmIL-2 for 3 days and then cell proliferation was assessed. It was found that CD4 T cells from control as well as 21 days p.i. animals had an increase in proliferation when treated with rmIL-2 together with the TCR stimulatory ligands (Fig 4C). In addition, we also evaluated GRAIL and Otub-1 expression as well as the phosphorylation of 4EBP1 in CD4 T cells with or without rmIL-2. In agreement with the increase in CD4 T cell proliferation from 21 days p.i., we found an increase in 4EBP1 phosphorylation and Otub-1 expression and a reduction in GRAIL expression (Fig 4D and 4E), indicating this E3 ubiquitin ligase to be a new player in T cell hyporesponsiveness during the acute phase of *T*. *cruzi* infection.

## Discussion

Several alterations of the immune response have been described in Chagas disease. Early investigations suggested that infection with *T*. *cruzi* was associated in both humans and mice with a severe T cell unresponsiveness to mitogens and antigens during the acute phase of the disease [37,38]. This immunosuppression was thought to facilitate the dissemination and establishment of the parasite in the infected host [9,39], which was ascribed to various mechanisms [5,8,40,41,42,43,44]. However, it has been widely demonstrated that the most affected cytokine in acute *T*. *cruzi* infection is IL-2, an important growth factor for T lymphocytes that is suppressed in several lymphoid organs such as thymus, mesenteric lymph nodes and spleen [45].

Many authors have shown that *T*. *cruzi* glycoproteins induce T cell anergy [37], cell cycle arrest [46] or inhibit T cell activation [5,6,8] by affecting IL-2 secretion or IL-2 receptor expression. In the present work, we found that CD4 T cells from acute *T*. *cruzi* infections in mice produced low levels of IL-2 when stimulated with anti-CD3/anti-CD28, and also had less capacity to proliferate, which could be related to the increase in GRAIL expression. In fact, the high expression of this gene alone is enough to convert a naïve CD4 T cell into an anergic phenotype [18]. Therefore, it is possible that T cell hyporesponsiveness caused by *T*. *cruzi* antigens such as mucins [8,37,46], and characterized by decreased IL-2 synthesis, might be mediated by GRAIL since expression of this E3 Ubiquitin Ligase correlates with the peak of the parasitemia. However, this still remains to be tested. Related to this, we have observed that GRAIL expression is induced directly by different *T*. *cruzi* strains.

It has been shown that parasite components such as mucins are able to inhibit early events in T cell activation and induce T cell anergy. These parasite components bind to L-selectin and inhibit different activation pathways that lead to inhibition of IL-2 secretion and T cell proliferation [8]. In addition, another work reported that parasite-derived mucins bind to Siglec-E (CD33) and inhibit mitogenic responses in CD4 T cells by inducing a cell cycle regulator that blocks the cell cycle [46]. It has also been shown that mannose-capped lipoarabinomannan (LAM) from *Mycobacterium tuberculosis* can inhibit CD4 T cell activation by downregulating the phosphorylation of key proximal TCR signaling molecules, which facilitates induction of anergy-related genes, and results in long-term CD4 T cell dysfunction [30]. In another study, engaging the TLR7 expressed on CD4 T cells resulted in complete anergy by inducing intracellular calcium flux, with the activation of an anergic gene-expression program being dependent on the transcription factor NFATc2. Then, T cell unresponsiveness was reversed by knockdown of TLR7 and restored the responsiveness of HIV-1+ CD4+ T cells *in vitro* [47]. Thus, it is possible that parasite derived factors might contact different receptors/molecules on T cells and induce T cell hiporesponsiveness directly by increasing the expression of anergy factors. We hypothesize that the high levels of parasites occurring during the acute phase of infection may induce GRAIL expression on T cells by a mechanism that we have not yet explored. However, additional studies are needed to understand how *T*. *cruzi* directly induces GRAIL expression on CD4 T cells.

Expression of the E3-Ubiquitin Ligase has been linked to CD4 T cell hyporesponsiveness during sepsis [29] and chronic murine schistosomiasis [31]. In addition, it has been reported to be induced by tegumental antigens from *Fasciola hepatica* and [28] LAM from *Mycobacterium tuberculosis* in CD4 T cells [30]. In fact, the T cell anergy observed during these infections is characterized by a lack of cytokine responses and reduced proliferative activity, which can be reversed by the addition of IL-2 and results in a reduction of GRAIL expression [29,30]. Although it has previously been shown that IL-2 is able to reverse the human T cell anergy induced by *T*. *cruzi* mucins [37], this is the first work that links the ability of IL-2 to reverse T cell hyporesponsiveness during *T*. *cruzi* infection to GRAIL regulation. During the acute phase of infection, we observed an increased expression of GRAIL with low Otub-1 and mTOR expression and activation in CD4 T cells. As Otub-1 is controlled by the Akt-mTOR pathway and is a negative regulator of the GRAIL function [19,25], this suggests that *T*. *cruzi* infection may disrupt the Akt-mTOR pathway resulting in Otub-1 downregulation, which in turn may induce GRAIL. In agreement with this, Sande *et al*. observed a downregulation of Otub-1 in LAM-treated T cells [30].

The absence of co-stimulation due to increased expression of inhibitory molecules such as CTLA-4 interferes with Otub-1 translation, with GRAIL expression being maintained via a blockade of the activation of the mTOR pathway [25]. In addition, most studies concerning T cell anergy have established that it results from TCR stimulation in an inhibitory environment, involving increased co-inhibition, decreased co-stimulation, or TCR engagement with a weak agonist peptide [48]. However, more recently, the role of mTOR and other related metabolic sensors and regulators has emerged as being of particular importance and has broadened our view of anergy-inducing signals [49,50]. Here, we observed differences in the expression of the co-inhibitory receptors PD-1 and CTLA-4 between CD4 T cells from acute and chronic phases of infection. An increased expression of CTLA-4 during the acute phase of infection may block mTOR activation, thus preventing protein translation, (including Otub-1) and leading to the maintenance of GRAIL expression with reduced T cell proliferation and cytokine production. Regarding this, we observed that CTLA-4 blockade in splenocytes from infected mice resulted in a reduction in GRAIL expression. Additionally, during several infections the expression of inhibitory molecules and E3 Ubiquitin ligases has been previously shown to be upregulated and to induce T cell hyporesponsiveness [51,52], with blocking CTLA-4 or neutralizing TGF-β during lymphatic filariasis restoring the ability to mount Th1/Th2 responses to live parasites and reversing the induction of anergy-inducing factors [51]. Furthermore, dendritic cells activated by tegumental antigens from *Fasciola hepatica* suppress T cells *in vitro* by inducing GRAIL and CTLA-4 expression [28]. Although the expression of inhibitory molecules has previously been observed during Chagas disease and in *T*. *cruzi* experimental infection [32,33,53], this is the first study that has demonstrated that CD4 T cell hyporesponsiveness may be caused by a combinatory effect of inhibitory molecules and GRAIL expression.

Considering the above results, we speculate that early upon infection parasite derived factors might contact receptors/molecules to induce directly GRAIL expression and T cell anergy. Later on, expression of inhibitory receptors such as CTLA-4 prevented Otub-1 protein expression and maintained GRAIL expression, which inhibited T cell proliferation. However, we have shown that GRAIL expression is reduced in T cells from acute infected mice cultured in the presence of CTLA-4 blocking antibodies or rm-IL-2. Therefore, these results may indicate that GRAIL expression during *T*. *cruzi* infection could be induced directly by the parasite and sustained by expression of inhibitory molecules.

GRAIL is not only expressed in naive T cells, but also in effector T cell subsets and controls their activation. Kriegel *et al*. reported that *GRAIL*-knockout Th1 effector CD4 T cells overproduce IFN-γ [17]. Related to this, we observed that CD4 T cells from acute infected mice, where GRAIL expression is increased, produced lower levels of IFN-γ than CD4 T cells from the later stages of *T*. *cruzi* infected mice. This is consistent with a recent report by Nunes *et al*. demonstrating that *in vivo* administration of *T*. *cruzi* mucin during murine experimental infection with *T*. *cruzi* parasites resulted in a lower number of splenic IFN-γ producing CD4 T cells [46], with these effects being accompanied by a greater susceptibility to infection, as shown by the higher levels of parasitemia [46].

CD3 is a known target of GRAIL, with the upregulation of GRAIL in T cells leading to degradation of CD3 [20]. In the present study, a lower CD3 expression in T cells was observed during the acute phase of infection that correlated with increased GRAIL expression (S1 Fig). Although additional experiments are needed to corroborate CD3-GRAIL interaction, it has been reported that immunosuppression during *T*. *cruzi* infection is due to defective T-Cell Receptor-CD3 functioning [54] and might be related to a lower expression of the CD3 molecule caused by degradation.

In summary, we have established a novel link between T cell hyporesponsiveness during *T*. *cruzi* infection and the expression and regulation of GRAIL in CD4 T cells. It is now important to extend these studies to evaluate additional GRAIL targets in *T*. *cruzi*-anergized T cells. In addition, it is necessary to determine if GRAIL can be detected in T cells from infected patients, and whether its expression can be induced directly in a dose dependent manner by purified antigens of *T*. *cruzi*. Finally, our data provide novel insights into why, despite the large immune cell activation by a wide variety of *T*. *cruzi* antigens, CD4 T cells may not respond optimally to their cognate antigens. In addition, T cell immune evasion strategies likely contribute to the host’s inability to eliminate *T*. *cruzi* and consequently permit survival and persistence of the parasite in the host.

## Supporting Information

S1 Fig Reduced CD3 expression in T cells during the acute phase of infection. Spleen cells isolated from control and infected animals were stained with fluorescence labeled anti-CD4 and anti-CD3 mAbs. Bars display CD3 mean fluorescence intensity (MFI) (n = 3, **p< 0.01, ***p< 0.0001, paired t- test versus control).
(TIF)
