# Supplementary figures and images for: GRAIL and Otubain-1 are Related to T Cell Hyporesponsiveness during Trypanosoma cruzi Infection
Source: PLoS Negl Trop Dis. 2017 Jan 23;11(1):e0005307. doi: 10.1371/journal.pntd.0005307 (PMC5289611; doi:10.1371/journal.pntd.0005307)

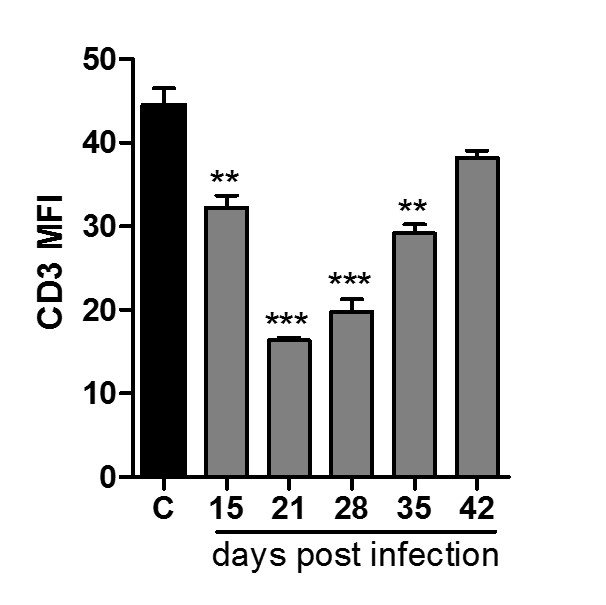

Supplement: S1 Fig — Spleen cells isolated from control and infected animals were stained with fluorescence labeled anti-CD4 and anti-CD3 mAbs. Bars display CD3 mean fluorescence intensity (MFI) (n = 3, **p< 0.01, ***p< 0.0001, paired t- test versus control). (TIF) [file pntd.0005307.s001.tif]
